# Effects of a social cue on reproductive development and pre-alternate molt in seasonally breeding migrant and resident female songbirds (*Zonotrichia leucophrys*)

**DOI:** 10.1242/jeb.160994

**Published:** 2017-08-15

**Authors:** Helen E. Chmura, Simone L. Meddle, John C. Wingfield, Thomas P. Hahn

**Affiliations:** 1 Department of Neurobiology, Physiology and Behavior, University of California, Davis, Davis, CA 95616, USA; 2 The Roslin Institute and Royal (Dick) School of Veterinary Studies, The Roslin Institute Building, The University of Edinburgh, Easter Bush Campus, Midlothian EH25 9RG, UK

**Keywords:** Migration, Reproduction, Song, Phenology, Avian, Seasonal timing

## Abstract

To time reproduction optimally, birds have evolved diverse mechanisms by which they respond to environmental changes that help them anticipate and prepare for the breeding season. While residents initiate reproductive preparation and breed in the same geographic location, migrant birds simultaneously prepare for breeding and migration far from their breeding grounds. As a result, it is hypothesized that migrant and resident birds use environmental cues differently to prepare to breed and that there is adaptive specialization in mechanisms regulating reproductive preparation. Specifically, residents are expected to rely more on non-photic cues (e.g. food, temperature, social cues) than migrants. We tested this general prediction using a social cue manipulation. First, we compared the effects of subspecies-appropriate recorded male song on reproductive development in migrants and residents on a naturally increasing photoperiod. Second, we tested the sensitivity of migrant-specific life history events (fattening and pre-alternate molt) to song treatment. After 82 days, residents had higher luteinizing hormone and greater ovarian development than migrants, but song treatment had no effect on these metrics in either subspecies. Song advanced pre-alternate molt but had no effect on fattening in migrants. While our study does not support specialization in social cue use in migrants and residents, it is consistent with findings in the literature of specialization in photoperiodic response. It also demonstrates for the first time that social cues can influence molt in a migrant species. Additional findings from a pilot study looking at responses to a live male suggest it is important to test other kinds of social cues.

## Abstract (precis)

**Highlighted Article:** Migrant and resident birds exhibit differences in reproductive development consistent with adaptive specialization in the use of photoperiodic cues, but not social cues, to regulate reproductive development.

## INTRODUCTION

For seasonally breeding vertebrates, the decision of when to breed can have dramatic fitness consequences. Selective environmental factors such as weather, food availability and predators can affect the optimal time frame for reproduction. Research suggests that differences in the timing of breeding can affect clutch size and offspring recruitment into the breeding population (Perrins, 1970; Verhulst and Nilsson, 2008). Given that timing of reproduction is so important, birds have evolved diverse reproductive schedules that match reproduction to fluctuations in conditions (Lofts and Murton, 1968). In different environments, reproduction can occur year-round (e.g. sooty terns; Chapin and Wing, 1959), seasonally (e.g. most temperate zone birds), or opportunistically in response to resource peaks (e.g. wild zebra finch response to rain; Zann, 1996). These variable reproductive schedules are coupled with complementary variation in timing of maintenance activities such as molt, as well as migration and dispersal, which also require careful timing decisions.

Seasonally breeding songbirds regulate the decision to breed on multiple time scales. In a theoretical framework described by Jacobs and Wingfield (2000) and Wingfield (2008), there is an initial preparatory period during which testes and ovaries begin to grow. This preparatory period is initiated by photostimulation of deep-brain photoreceptors (Halford et al., 2009; Nakane et al., 2010) which initiate a cascade of physiological changes that lead to the release of gonadotropin-releasing hormone (GnRH) and an increase in circulating luteinizing hormone (LH) and follicle stimulating hormone (FSH) (Dawson et al., 2001). This initial developmental period is followed by mature expression of the breeding life history stage, when birds build nests, copulate, lay eggs and rear young. The environmental cues that birds use to regulate each phase of breeding may differ. Initial predictive cues, such as photoperiod and endogenous circannual rhythms, initiate the developmental phase and drive gonadal recrudescence. For some species, these cues must be perceived before animals can respond to other kinds of information. Local predictive cues, such as weather and food availability, can accelerate or inhibit gonadal development and mature expression of breeding to match physiology to local environmental conditions. Social cues (also called synchronizing or integrating cues) are also thought to influence reproductive development after the perception of initial predictive cues, and ensure that the timing of events is synchronized across a social group (e.g. Perfito et al., 2015).

Variation in reproductive timing may result from variation in regulatory mechanisms, variation in environmental conditions or both processes acting on present day and evolutionary time scales (Hahn and MacDougall-Shackleton, 2008). Hahn and MacDougall-Shackleton (2008) provide a framework that offers three broad hypotheses as to how variation in timing of seasonal changes in behavior and physiology may arise. The ‘adaptive specialization’ hypothesis proposes that organisms' cue–response systems have evolved to adaptively match variation in environmental conditions and this mechanistic diversity gives rise to variation in timing of seasonal events. The ‘conditional plasticity’ hypothesis proposes that organisms' cue–response systems can create numerous behavioral timing outcomes, and that much observed variation in behavioral timing is the result of plastic responses to environmental variation and not mechanistic diversity. The third hypothesis focuses on phylogenetic history and suggests that variation in seasonal timing may be the byproduct of mechanisms shaped by prior evolution and may be non-adaptive or neutral in the present environment.

Migratory birds, which exhibit predictable seasonal movement patterns, are thought to use different environmental cues from those used by residents to prepare to breed. Migrants must initiate reproductive preparation in a location that may be separated by thousands of kilometers from their breeding grounds, whereas residents prepare close to where they actually breed. For this reason, theory predicts that the cue–response systems of residents may respond more (or earlier) to local weather and social cues than those of migrants even during gonadal growth. In contrast, migrants are thought to rely primarily on photoperiodic and/or circannual rhythms (Both and Visser, 2001; Gwinner, 1996; Wingfield et al., 1992) and have limited gonadal response to other kinds of information. This is consistent with the adaptive specialization hypothesis. However, because migrants and residents of the same subspecies often breed in different locations, it can be difficult to demonstrate that differences in gonadal development are indicative of adaptive specialization in cue–response systems and not a result of being exposed to different cues. Controlled experiments are necessary to distinguish between variation in seasonal timing that is the result of adaptive specialization in cue–response mechanisms and variation which is driven by plasticity that is conditional upon environmental variation.

There is a rich tradition examining variation in how photoperiod is used as a cue regulating life history transitions (e.g. Ball, 1993; Farner et al., 1993; Hau et al., 1998; Nicholls et al., 1988). Much is known about intraspecific variation in response to photoperiodic cues (Lofts and Murton, 1968), and some studies show that migrants and residents within the same species have different responses to increasing spring photoperiod (e.g. white-crowned sparrows: Ramenofsky et al., 2017; dark-eyed juncos: Fudickar et al., 2016; stonechats: Helm, 2009; but see Atwell et al., 2014; Perfito et al., 2005). Variation in how non-photic cues are used across or within taxa, and in particular between migrants and residents, is less understood. Previous studies suggest that migratory strategy is associated with variation in the use of temperature cues, with short-distance migrants being more responsive to temperature cues than long-distance migrants when tested on breeding ground photoperiods (Wingfield et al., 2003, 1996, 1997; but see findings in altitudinal migrants in Perfito et al., 2005). However, it is difficult to disentangle variation due to migratory distance from that due to breeding latitude, as longer distance migrants often breed at higher latitudes with longer photoperiods, and latitudinal variation in temperature cue–response systems has also been shown in populations of resident great tits (Silverin et al., 2008). Research also indicates that there is taxonomic variation in response to food cues (Schoech and Hahn, 2007). Again, studies find an association between food cue use and breeding latitude (e.g. Schoech and Hahn, 2007); however, the association between this variation and migratory strategy is untested**.** Other kinds of non-photic cues including water availability (Perfito et al., 2006; Wingfield et al., 2012) also influence reproductive development.

The relationship between social cues and reproductive development may be particularly complex (e.g. Bentley et al., 2000; Brockway, 1965; Kroodsma, 1976; Stevenson et al., 2008) and recent reviews call for better integration of research on timing mechanisms and social behavior (Helm et al., 2006). Although research relating the use of social cues in reproductive preparation to migratory strategy is non-existent, studies suggest that they play a crucial role in reproductive preparation, often in sex- and time-dependent ways. For example, in pine siskins, female reproductive development is sensitive to hormonal manipulations of their partners, while development in males is unresponsive to female hormonal manipulations (Watts et al., 2016). In contrast, Runfeldt and Wingfield (1985) found that hormonal manipulations of a partner extend the reproductive period in male but not female birds. Social cues may be particularly important during final but not early stages of starling reproductive preparation (Perfito et al., 2015) and may also modify the effects of other kinds of information. For example, female Puget Sound white-crowned sparrows accelerate reproductive development in response to high temperatures only when housed with males (Wingfield et al., 1997).

While adaptive specialization of cue use in migrants and residents remains a cornerstone of many ecological theories, it remains largely untested. Experiments that place closely related subspecies in laboratory conditions that manipulate exposure to environmental cues play an important role in testing whether differences in behavioral timing observed in the field are the result of differences in cue–response mechanisms (consistent with the adaptive specialization hypothesis) or arise from environmental variation (consistent with the conditional plasticity hypothesis). In this study, we tested the hypothesis that birds with different life histories will regulate the developmental phase of gonadal growth differently in response to non-photic cues. We tested this broad hypothesis within the specific context of social cue use by comparing the response of migrant and resident female white-crowned sparrow subspecies to a subspecies-appropriate recorded male song cue. If the birds exhibited specialization in social cue use, we predicted that resident birds would advance reproductive development more than migrants in response to a recorded male song cue. If migrants and residents exhibited no differences in song response (either both subspecies responding or both not responding), this would suggest that specialization in social cue–response mechanisms does not influence differences in migrant and resident reproductive preparation. In migrants, we also tested the response of additional pre-breeding spring life history stages, migratory fattening and pre-alternate molt (Humphrey and Parkes, 1959), to recorded male song cues. While residents do not exhibit molt or pre-migratory fattening, making them inappropriate metrics for testing sub-specific variation in cue–response systems, we collected these data in migrants to gain a better understanding of how social cues may influence regulation of multiple life history stages across the annual cycle.

## MATERIALS AND METHODS

### Birds

Gambel's white-crowned sparrows [*Zonotrichia leucophrys gambelii* (Nuttall 1840)] and Nuttall's white-crowned sparrows (*Zonotrichia leucophrys nuttalli* Ridgway 1899) are closely related subspecies (Weckstein et al., 2001). Despite their shared lineage, they have divergent life history strategies. Gambel's white-crowned sparrows (hereafter migrants) are long-distance migrants, spending the wintering months in California, southwestern USA and Mexico before migrating up to 3000–4000 km to breed in northern Washington, Alaska and western Canada (Chilton et al., 1995). In contrast, Nuttall's white-crowned sparrows (hereafter residents) have a sedentary life history and live on the coast of northern California year round (Chilton et al., 1995). Additional differences in life history are associated with these two subspecies' divergent migratory strategies. In spring, migrants undergo a pre-alternate molt, replacing body feathers and central rectrices, and a period of hyperphagia and muscle hypertrophy. In contrast, pre-alternate molt is rare and of limited extent in residents and changes in muscle and fat are absent (Ramenofsky et al., 2017). Initiation of breeding occurs at different times of the year: residents may begin to breed as early as March or April (Blanchard, 1941; Mewaldt and King, 1977), while migrants do not arrive on the breeding grounds and begin nesting until May (Norment, 1992). The divergent life history strategies of migrant and resident white-crowned sparrows make them an ideal system in which to study variation in the seasonal timing of regulatory mechanisms with minimal phylogenetic confounds.

### Field capture and acclimation to captivity

Juvenile female migrant and resident sparrows were caught using baited potter traps and mist-nets between 6 November and 18 December 2015 in Sonoma and Yolo Counties, CA, USA. At the time of capture, birds were initially sexed using wing-length measurements; however, a few males were identified during initial laparotomies (see below) and removed from the study. During an initial period of acclimation to captivity, birds were held in same-subspecies groups of 10–20 individuals in indoor flight aviaries (L×H×W: 2.7×2.7×1.2 m^3^) on the naturally changing photoperiod of Davis, CA, USA (39°N), controlled by a Paragon EL72PC digital timer. On 13 January 2016, birds were transferred to individual cages (L×H×W: 38.1×45.7×35.5 cm^3^) in sound attenuation chambers or ‘minibooths’ (IAC Acoustics, North Aurora, IL, USA) randomized by capture date. For identification, all birds were given individual numbered plastic leg bands. Chambers contained six birds each with two birds per shelf and within each chamber all birds were in acoustic contact with each other. Birds on the same shelf could see each other. During this acclimation period, initial laparotomies were performed to verify sex (see below), and birds were randomly assigned to male song or no male song treatment groups. Initial sample size was *n*=12 for each migrant and resident song treatment groups, *n*=12 for the resident control group and *n*=6 for the migrant control group.

During the course of the experiment, it was determined that an additional chamber of six birds initially assigned to the migrant control group actually contained a male bird. The birds in this group (*n*=5) were re-assigned to a live male treatment group and were analyzed separately.

While in chambers, photoperiod was increased once per week to match the naturally increasing photoperiod of 39°N for the duration of the experiment using a digital timer. Chambers were lit with 40 W natural spectrum (6500 K) bulbs (Verilux Inc., Waitsfield, VT, USA). During both the acclimation and treatment phases of the experiment, birds received *ad libitum* access to water, romaine lettuce, Mazuri Small Bird Maintenance Mini Diet (Richmond, IN, USA), and sand for grit. All work with live birds was done in accordance with UC Davis IACUC protocol no. 19029, USFWS Permit MB813248, California State Permit SC-000519, and a California State Parks Permit for Sonoma County, and the number of birds used was minimized.

### Treatment

Treatment with song or no song was initiated on 4 February 2016 (Julian date 35). For the next 82 days, birds in song treatment groups were exposed to 4 h per day of audio recordings of subspecies-appropriate male song beginning at lights on. Songs were selected to mimic the diversity of songs that birds would hear on the breeding grounds. There is tremendous variation in migrant male song within one breeding locality (DeWolfe et al., 1974; Madeline and Handford, 1991), and while there is some evidence to suggest there could be large-scale patterns linking geographic variation and song, playback studies do not suggest that females discriminate between songs recorded locally and at distance (Nelson, 1998). As such, migrant song recordings included exemplars from 10 unique individuals on the breeding grounds, representing five song types with two representatives from different individuals per type (recordings courtesy of the Macaulay Library of Natural Sounds at the Cornell Laboratory of Ornithology). In contrast, resident sparrows have regional song dialects on the breeding grounds (Baptista, 1975) and there is some evidence that resident female birds exposed to their natal dialect engage in more nest building activities than those exposed to alien dialects (Spitler-Nabors and Baker, 1983). As such, resident song recordings included exemplars from 10 unique individuals recorded during breeding at Sonoma Coast State Park, where birds were caught, generously provided by Elizabeth Derryberry of Tulane University (New Orleans, LA, USA). All recordings were standardized to identical peak amplitude using Raven Pro 1.4 software (http://www.birds.cornell.edu/brp/raven/RavenVersions.html) before assembling them into sequences for playback. To create biologically appropriate playback sequences, one song exemplar per individual was repeated 5 times per minute, which is the song rate of males in nature, to create one song bout. Song bout recordings were set to play in a random order; however, each day birds would hear song bouts from each individual 24 times. Songs were played in .wav file format using MP3 players and mini-speakers (JBL by Harman, Stanford, CT, USA). Song amplitude was measured with an SPL Meter System 824 (Larson Davis, Depew, NY, USA) and varied from 68 to 85 dBA with cage position in the chamber and distance to speaker. Throughout the experiment, onset of morning song was shifted earlier once per week to match lights on and the changing time of dawn chorus that birds would experience in the field.

As noted above, one separate group of migrant birds (*n*=5) were assigned to a live male treatment group in which they had auditory contact (but no tactile and limited visual contact) with a live male housed in the same chamber.

### Measurements

Throughout the experiment, measurements of body mass, molt progression and ovarian development were made. Mass was measured with birds in a small mesh bag hung from a Pesola scale. Mass was analyzed as a scaled mass index to correct for mass differences attributable to structural size differences between individuals (Peig and Green, 2009). Mass index was calculated using skull length and initial wild capture mass to generate the scaling factor *b*_SMA_. Molt progression was measured in three body regions (crown, abdomen, back) on an ordinal scale of 0–3 with 0 representing no molt and 3 representing heavy molt (more than 50% of feathers being grown) (Ramenofsky and Németh, 2014). A total molt intensity score was created by summing scores from the three body regions.

Reproductive development was assessed multiple ways. Biweekly blood samples were taken to track changes in hormones associated with reproductive preparation. All samples were taken within 3 h of lights-on. During sampling, each cage and each chamber was sampled in a random order and blood was collected within 10 min of opening the chamber door. Blood samples were collected from the alar vein using a 26 gauge sterile needle and heparinized capillary tubes. After collection, blood was stored on ice until it was centrifuged at 12,000 ***g*** for 5 min. Plasma was aspirated from the capillary tubes with a Hamilton syringe and stored in labeled Eppendorf tubes at −30°C until hormone assays were conducted. At the conclusion of the experiment, trunk blood was collected after birds were killed using heparinized capillary tubes (see below) and was processed as above.

Ovarian development was assessed during monthly laparotomies (one baseline measure, two mid-experiment measures, and one terminal measure). For this procedure, birds were anesthetized using isoflurane (Piramal, Bethlehem, PA, USA) gas delivered with a Summit Anesthesia Vaporizer (Bend, OR, USA) at a dose of 3–5%. When the bird was sedated, a small (∼1 cm) incision was made on its left side just below the lowest rib through which the ovary and developing follicles could be observed. Ovarian development was scored on an ordinal scale from 1 to 6 as: 1, a smooth completely regressed ovary with no visible follicles; 2, a granular ovary; 3, visible follicles without hierarchy; 4, follicles with a hierarchy but no yolky follicles; 5, hierarchical follicles beginning to yolk; and 6, an egg in the oviduct (Hahn, 1998). Incisions were sealed with surgical adhesive (MWI, Meridian, ID, USA) and birds were given an intramuscular (pectoralis) injection of 0.1 mg ml^−1^ meloxicam (MWI) in sterile saline at a dose of 0.5 mg kg^−1^ as an analgesic. Birds were returned to cloth bags to recover, and as soon as anesthesia had worn off (typically in 5 min), they were returned to individual cages and monitored for 7 days for adverse effects (none were observed). All laparotomies were performed by the same investigator who was blind to bird treatment.

At the termination of the experiment, birds were killed with an overdose of isoflurane gas followed by rapid decapitation and trunk blood was collected. Terminal ovarian and oviduct mass measures were made by dissecting tissues and weighing to the nearest milligram. The size of the largest follicle was measured to the nearest 0.5 mm using dial calipers. Ovarian development was also scored as during laparotomies (see above), and tissues (liver and brain) were preserved for future studies.

### Hormone assays

Biweekly blood samples were analyzed for LH in duplicate in a single radioimmunoassay using a protocol modified from Sharp et al. (1987). Briefly, 20 μl of plasma or standard, 20 μl of primary rabbit anti-LH antibody and 20 μl of I^125^-labeled LH were combined. After incubation overnight at 4°C, 20 μl donkey anti-rabbit precipitating serum with 20 μl of normal rabbit serum was used to separate bound versus free I^125^ LH label. The intra-assay variation (calculated from the coefficient of variation from samples where both duplicates had detectable hormone levels) was 20.8% and the minimum detectable concentration of LH was 0.31 ng ml^−1^. This assay has been used to quantify circulating LH levels previously in white-crowned sparrows (Wingfield et al., 2003, 1996, 1997).

Trunk blood samples were analyzed for 17β-estradiol (E_2_) in duplicate in a single radioimmunoassay. Briefly, a plasma volume of between 50 and 200 μl was combined with deionized water to bring it up to a total volume of 400 μl. To assess extraction efficiency, 20 μl of ^3^H-E_2_ (2000 cpm, NET-517, PerkinElmer, Waltham, MA, USA) was added and samples were incubated at 4°C overnight to equilibrate with steroid binding proteins. Steroids were extracted from samples with 4 ml of diethyl ether for 1 h, at which point ether was decanted and dried under N_2_ in a 35°C water bath. Samples were reconstituted in 550 μl phosphate-buffered gelatin saline (PBSG) and gently shaken for 3 h; 200 μl sample duplicates and 100 μl sample recoveries were aliquoted. Each duplicate received 100 μl ^3^H-E_2_ (10,000 cpm) and 100 μl of 1:500 dilution antibody (CAT ABIN 1826595, Antibodiesonline.com, Atlanta, GA, USA) and refrigerated at 4°C overnight. Antibody validation is described below. Dextran-coated charcoal in PBSG was used to separate unbound from bound hormone, after which samples were incubated at 4°C for 12 min and then centrifuged at 12,000 rpm at 4°C for 10 min. Samples were decanted into a scintillation vial and suspended in 3 ml Ultima Gold scintillation fluid (PerkinElmer, Waltham, MA, USA). Samples were counted for 4 min in a Beckman Coulter LS6500 counter (Indianapolis, IN, USA). The intra-assay variation was 4.23% and the detection limit was 2.04 pg per tube.

### Antibody validation

Antibody validation for the LH assay was as described previously (Sharp et al., 1987; Wingfield et al., 1996, 1997, 2003). For the E_2_ assay, antibody was validated for focal species by checking for parallelism between standard curves of serially diluted E_2_ standard (E8875, Sigma-Aldrich, St Louis, MO, USA) and separate plasma pools of resident and migrant white-crowned sparrows that had been stripped of endogenous hormone with dextran-coated charcoal and spiked with E_2_ standard.

### Statistical analyses

All data were analyzed in R (version 3.2.4; http://www.R-project.org/) with packages lme4 (Bates et al., 2015), lmerTest (version 2.0–20; https://CRAN.R-project.org/package=lmerTest) and ordinal (version 2015.6-28; https://CRAN.R-project.org/package=ordinal). One bird died during the course of the study and data from that individual were only available to Julian date 90. Three birds were excluded from the final analysis: one resident and one migrant were excluded because of health concerns (e.g. weight loss) during the course of the study, and one bird was excluded because its morphology was deemed to be more like that of a third closely related subspecies, the Puget Sound white-crowned sparrow, which is known to interbreed with residents (Corbin and Wilkie, 1988).

All models were built with full random effects structures (Barr et al., 2013) including shelf (cage position within chamber) and, for repeated measures, individual ID nested within shelf position. We *a priori* chose to include fixed effects for subspecies, song treatment and a subspecies by song treatment interaction for all single time point outcomes and added time and all additional two- and three-way interactions for repeated measures outcomes. In our analysis of migrant birds exposed to a live male, we made comparisons both with migrants exposed to song treatment and control birds. Ordinal variables (laparotomy score and molt score) were modeled using the clmm function in ordinal and continuous measures (LH levels, terminal tissue measures) were modeled using the lmer function in lme4 with maximum likelihood estimation. After full models were fitted, non-significant terms were dropped and a reduced model was constructed and compared with the initial full model (Zuur et al., 2009). This process was repeated until dropping additional terms made model performance decline (Zuur et al., 2009). If an optimal model could not be distinguished (i.e. multiple models performed similarly), the full model results for the non-null model with the lowest Akaike's information criterion (AIC) score and fewest number of predictors is reported. In all such cases, the statistically significant terms between similarly performing models were the same. A summary of model selection results is reported in Table S1. Normality and variance of residuals for the optimal model were inspected visually. Selected within-time point *post hoc* comparisons were conducted to explore complex interactions revealed in initial models for follicle stage score and mass index. Given that shelf was a non-informative parameter and interfered with model convergence with small sample sizes, it was not included as a random effect for follicle stage score *post hoc* tests. Similarly, as not all groups contained multiple chambers, it was not possible to include chamber as a random effect in the model. However, in an analysis of data from resident groups only (for which multiple chambers were present in both song and no-song groups), very little variation in response was attributed to chamber, suggesting that chamber effects are probably not important. Additionally, as terminal organ measures could not be scaled, supplementary analyses looking at the effect of a structural size measure (head) and a structural size by subspecies interaction on terminal organ mass was conducted to ensure that structural size differences were not driving observed patterns.

## RESULTS

### Effects of song treatment on reproductive readiness in migrants and residents

**Fig. 1.**
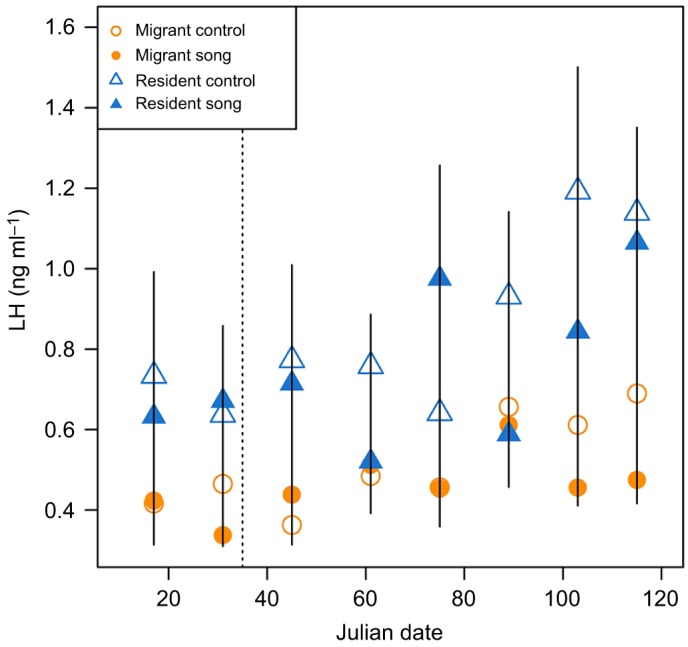
**Effects of song treatment on circulating plasma luteinizing hormone (LH).** Migrants (circles) and residents (triangles) were exposed to song (filled symbols) or control no-song (open symbols) treatments. Data are presented as means±s.e.m. and the vertical dashed line represents the Julian date that song treatment began.

LH secretion was higher in residents than in migrants (β=0.315, *t*=3.378, *P*=0.002; Fig. 1; Table S2), and the change in LH increased over time in both subspecies (β=0.003, *t*=3.794, *P*<0.001). Inclusion of terms for song treatment and all 2- and 3­-way interactions did not improve model performance (Table S1).

**Fig. 2.**
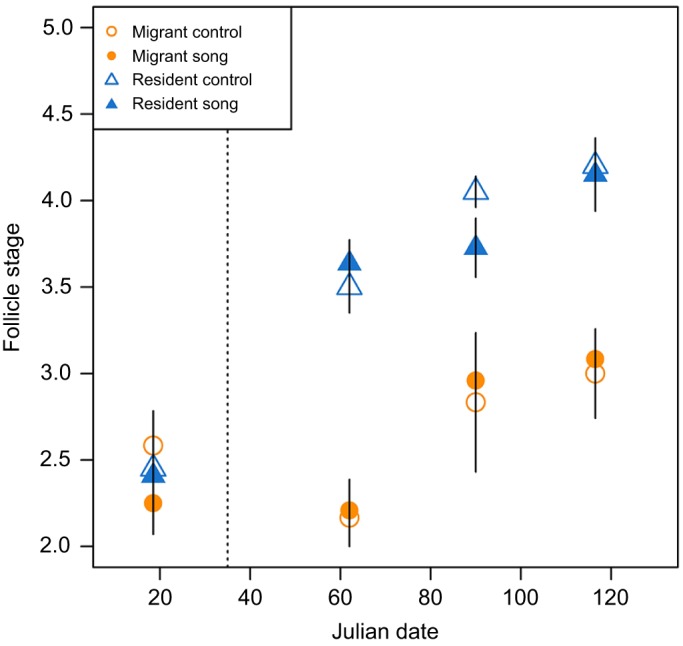
**Effects of song treatment on follicle stage.** Follicle stage measured by laparotomy was measured in migrants (circles) and residents (triangles) exposed to song (filled symbols) or control no-song (open symbols) treatments. Follicle stage is depicted as mean±s.e.m. for graphical display but was treated as an ordinal variable in all analyses. The dashed line represents the Julian date that treatment began.

Model estimates showed that the probability of receiving a high follicle stage score increased more over time in residents than in migrants (β=0.062, *t*=4.150, *P*<0.001; Fig. 2; Table S2). Although the overall model picked up a significant effect of a 3-way interaction between subspecies, time and song, *post hoc* tests show that song treatment or a song treatment by subspecies interaction did not significantly explain differences in follicle stage score at any individual sampling point (Table S3). On the pre-treatment sampling date, there were no differences in follicle size score between subspecies (β=−0.522, *t*=−0.546, *P*=0.585), song treatment groups (β=−1.046, *t*=−1.087, *P*=0.277) or their interaction (β=1.266, *t*=0.996, *P*=0.319). However, at each subsequent sampling point, residents had higher follicle size scores than migrants: Julian date 66 (β=5.811, *t*=3.580, *P*<0.001), Julian date 90 (β=4.673, *t*=3.401, *P*<0.001) and terminal sample (β=5.543, *t*=3.871, *P*<0.001).

Ovary mass (β=0.015, *t*=2.54, *P*=0.015; Table S2) and follicle size (β=1.291, *t*=4.187, *P*<0.001; Table S2) were significantly higher in resident birds than in migrants and there was a trend in the same direction for larger oviduct mass in residents (β=0.115, *t*=1.9143, *P*=0.06; Table S2). In an alternative analysis, accounting for the potential influence of structural size, predictors of structural size, subspecies and their interaction did not significantly predict ovary mass, oviduct mass or follicle size (Table S4). However, model comparison suggests that model performance for ovary mass and follicle size improved with the inclusion of structural size predictors over a subspecies-only model. Inclusion of song treatment as a predictor did not improve model performance (Table S2).

Terminal E_2_ levels were undetectable in all but four sampled birds (one per study group). As a result, no statistical analyses of these data were possible.

### Effects of song treatment on morphology in migrants and residents

**Fig. 3.**
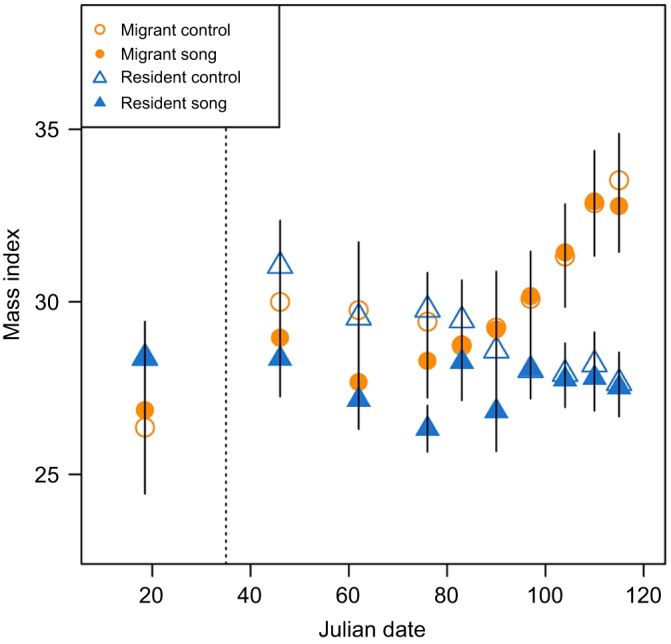
**Effects of song treatment on body mass.** Scaled mass index (*b*_SMA_) was measured in migrants (triangles) and residents (circles) exposed to song (filled symbols) and control no-song (open symbols) treatments. Data are displayed as means±s.e.m. Vertical dashed line represents the Julian date that song treatment began.

Mass index changed over time (β=0.057, *t*=0.005, *P*<0.001; Fig. 3; Table S2); however, there was a significant time by subspecies interaction (β=0.071, *t*=0.007, *P*<0.001). *Post hoc* tests show that while mass index was the same in both subspecies at the initial pre-song treatment sampling date (β=1.673, *t*=1.741, *P*<0.089; Table S4), at the terminal post-treatment sampling date, mass index was significantly lower in residents than in migrants (β=−5.442, *t*=−5.005, *P*<0.001). Inclusion of song treatment did not improve model prediction (Table S1).

### Effects of song treatment on molt in migrants

**Fig. 4.**
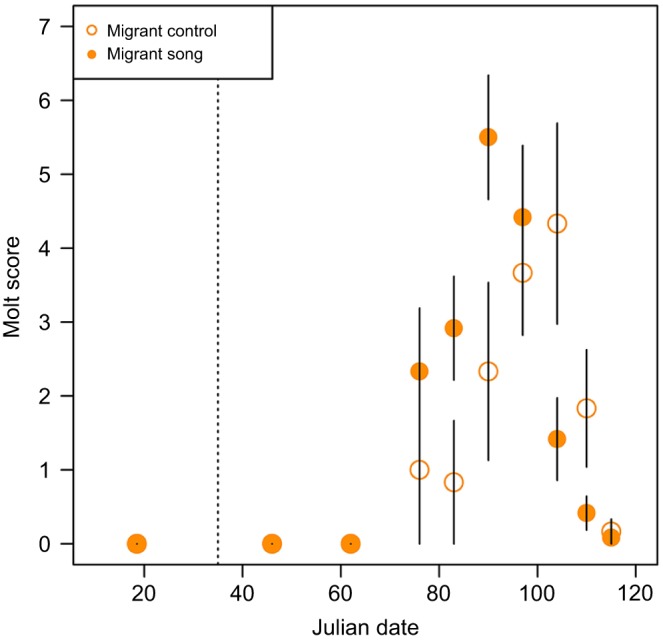
**Effects of song treatment on molt in migrants.** Molt score in migrants exposed to song (filled circles) or control no-song (open circles) treatments over time. Molt score is depicted as mean±s.e.m. for graphical display but was treated as an ordinal variable in all analyses. The dashed line represents the Julian date that song treatment began.

Molt dynamics differed between control migrants and migrants exposed to song (Fig. 4; Table S2). Peak molt occurred earlier and terminated sooner in song-exposed migrants than in control migrants. On the day that the largest average molt score occurred in song-exposed migrants (Julian date 90), there was a trend for a higher molt score in these birds than in control birds (β=1.743, *z*=1.836, *P*=0.066). On Julian date 104, the day that the largest average molt score occurred in control migrants, molt score was significantly higher in these birds than in song-exposed birds (β=−2.081, *z*=2.146, *P*=0.0319). Models including song treatment outperformed null models (Table S1). Given that the extent of pre-alternate molt is extremely limited in resident birds, we did not test differences between song treatment groups in this subspecies.

### Effects of live male on reproductive readiness in migrants

**Fig. 5.**
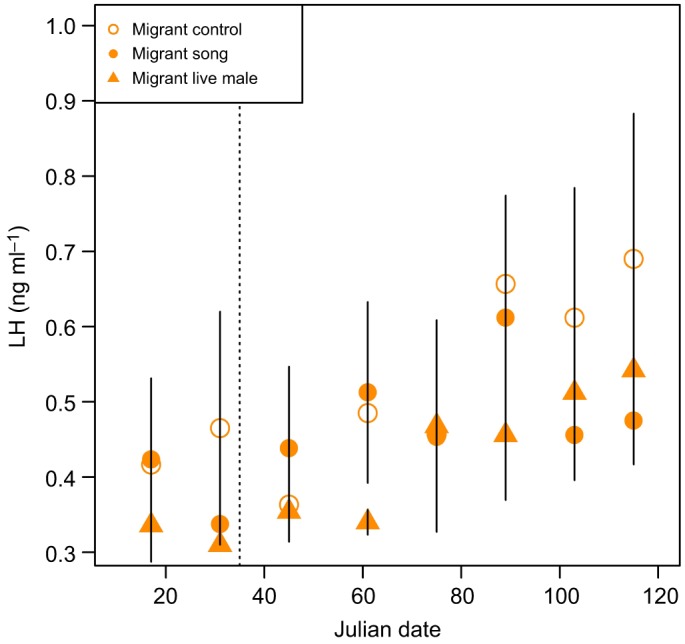
**Effects of song or live male treatment on circulating LH in migrants.** Migrants exposed to control no-song (open circles), song (filled circles) and live male (filled triangles) treatments. Data are presented as means±s.e.m. and the dashed vertical line represents the Julian date that song treatment began.

**Fig. 6.**
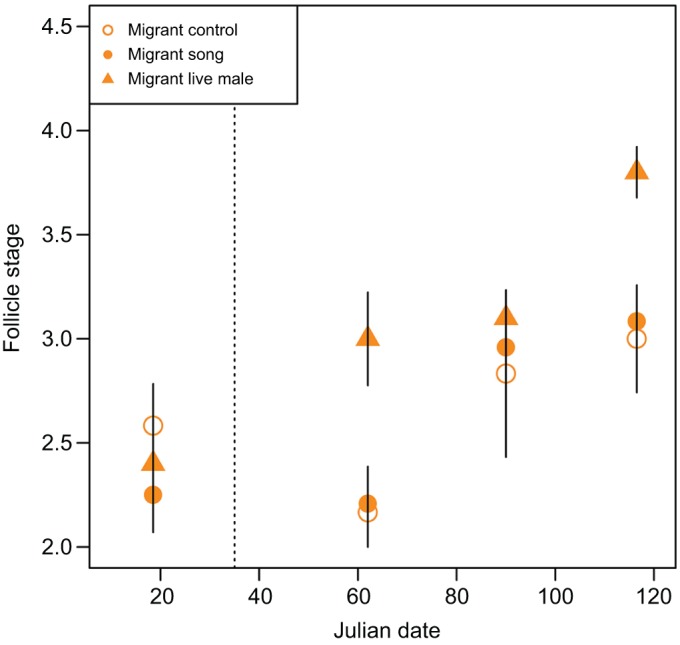
**Effects of song and live male treatment on follicle stage in migrants.** Follicle stage score was measured by laparotomy in migrants exposed to control no-song (open circles), song (filled circles) or live male (filled triangles) treatments. Follicle stage is depicted as mean±s.e.m. for graphical display but was treated as an ordinal variable in all analyses. The dashed line represents the Julian date that song treatment began.

**Fig. 7.**
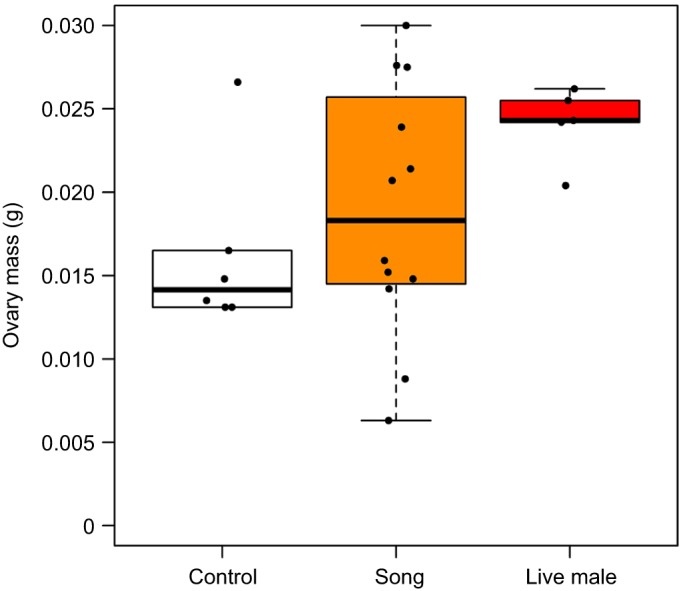
**Effects of song and live male treatment on ovary mass in migrants.** Terminal ovary masses in migrants exposed to control no-song, song or live male treatment are represented as boxplots. Boxes represent the median, 25th and 75th percentile values. Error bars represent these percentiles ±1.5 times the interquartile range, except for cases in which the largest observed data point is smaller than this value, or the smallest observed data point is larger than this value. Points represent individual birds.

**Fig. 8.**
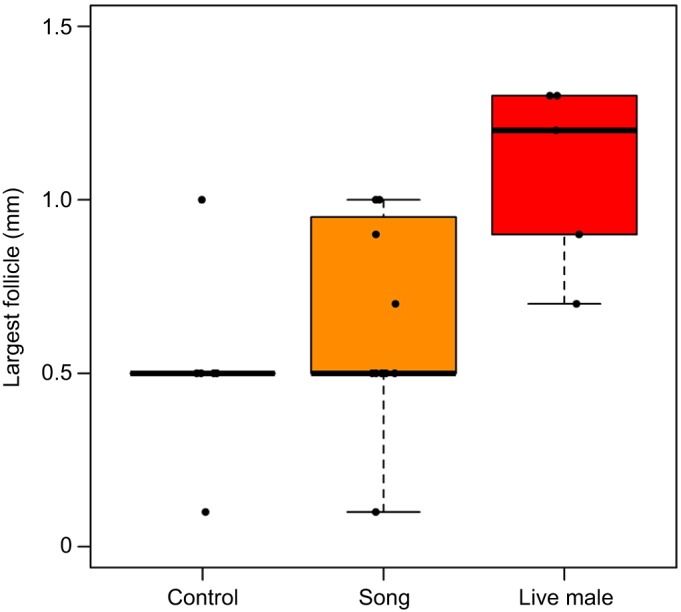
**Effects of song and live male treatment on follicle size in migrants.** Terminal sizes of the largest follicles in control no-song, song and live male treated birds are depicted as boxplots. Boxes represent the median ± the 25th and 75th percentile of data. Error bars represent ±1.5 times the interquartile range. Points represent individual birds.

LH increased over time (β=0.002, *t*=3.581, *P*<0.001; Fig. 5; Table S2) in all birds regardless of exposure to a live male, song treatment or control treatment. In contrast, birds exposed to a live male increased follicle stage score more over time than birds in the control group (β=0.034, *z*=0.017, *P*=0.039) (Fig. 6; Table S2), while there was no difference in how follicle stage score changed over time between birds exposed to song treatment and the control group (β=0.018, *z*=1.262, *P*=0.207). For two of three terminal measures, the reproductive organs of migrant birds exposed to a live male showed greater maturity than birds in the control group: ovary mass (β=0.008, *t*=2.316, *P*=0.031; Fig. 7; Table S2) and follicle size (0.563, *t*=2.703, *P*=0.0127; Fig. 8; Table S2). However, oviduct mass was not different between live male and control treatments (β=0.003, *t*=1.386, *P*=0.181), and the intercept-only model had a slightly lower Akaike's information criterion (AIC) score than the model with treatment predictors. Consistent with the analyses conducted between subspecies, the analysis restricted to migrants also found no differences in terminal measures between migrants with or without exposure to song.

## DISCUSSION

Our results suggest that a recorded male song cue had no effect on hormone secretion or gonadal recrudescence in either migrant or resident females. This runs contrary to our predictions and does not support the hypothesis that residents would be more responsive to song cues than migrants. Residents advanced gonadal development more than migrants but song cue did not cause detectable changes in gonadal recrudescence in either subspecies. This is consistent with common garden studies suggesting that migrants and residents may respond differently to photoperiodic cues. Thus, while we do not find evidence for specialization in social cue response between migrants and residents, our results are consistent with literature suggesting specialization in photoperiodic response. Our study also suggests that other life history stages might show different sensitivity to social cues and that different kinds of social cues might elicit stronger gonadal responses. Migrants exposed to recorded song stimuli advanced pre-alternate molt compared with controls. Additionally, migrants exposed to a live male exhibited increased gonadal development. These offer promising future avenues for studying differences in cue use across life history stages.

### Gonadal response to male song cue

The lack of a reproductive response to song in both subspecies, which occurred at the level of circulating LH and at the level of the gonad, is surprising. Numerous previous studies have used a song-recording experimental paradigm with observable effect on ovarian development (Bentley et al., 2000; Brockway, 1965; Hinde and Steel, 1978; Kroodsma, 1976; Leboucher et al., 1998; Morton et al., 1985). In designing song treatment, we were careful to address aspects of song selection (e.g. avoiding pseudoreplication and choosing subspecies-appropriate dialectical and song-type variation) and song administration (time of day, amplitude, random ordering) that are thought to affect species' response to song cues. Therefore, our song treatment should have been sufficient to elicit a gonadal response if such an effect exists in response to recordings.

One potential explanation for the difference between our study and others is that song cues are most important during later stages of reproductive development. It is possible that initial ovarian growth immediately following photostimulation may be relatively insensitive to song, but later phases of ovarian growth (e.g. yolk deposition and final follicle maturation) may be more sensitive. The importance of social interactions with a male during the final phase of follicular maturation has been shown elegantly in starlings (Perfito et al., 2015). However, previous work in migrants by Morton et al. (1985) found that song augmented ovarian growth even during initial ovarian growth, but that the effects of song depended upon photoperiod. Ovarian growth rate constants were augmented by song at 12.5 and 14 h light, but not at shorter or longer photoperiods. A similar interaction between photoperiod and song response has been reported in other species (Hinde and Steel, 1978). In our study, birds experienced day lengths longer than 12.5 h light for a minimum of 30 days in naturally increasing photoperiod conditions, which should be sufficient to allow a song treatment effect to emerge. Instead, differences between our study and work by Morton et al. (1985) may be explained by differences in outcome measures; Morton et al. (1985) found differences between treatment groups in ovarian growth rate constants (a measure calculated from terminal ovarian mass measurements at every sampling date) and only report significant differences in ovarian mass at the 14 h light photoperiod. Given that we could not measure ovarian mass until the termination of our study, it was not possible to calculate a growth rate constant or comparable metric.

Alternative explanations for our findings emphasize the importance of considering the nature of social cues chosen for such experiments. It is possible that a more robust response to song treatment would be observed with other kinds of social cues. Songs were selected as representative of the breeding grounds, but if there were important differences in adult song between breeding and wintering locations (DeWolfe et al., 1974; Meitzen et al., 2009), this temporal variation in song, rather than song per se could be the relevant biological cue. Previous studies have shown that small differences in song can cause differences in ovarian response. Conspecific male song stimulates ovarian response more than heterospecific song (Bentley et al., 2000), larger repertoire song treatments are more stimulatory than smaller repertoire treatments (Kroodsma, 1976), and physiological responsiveness may be enhanced in response to native rather than alien dialect types (Spitler-Nabors and Baker, 1983; but see MacDougall-Shackleton et al., 2001). Additionally, specific components of the vocal repertoire may enhance ovarian development (Brockway, 1965). Our finding that migrants enhanced gonadal development when exposed to a live male compared with the control (no-song) birds, but that there were no differences between migrant birds exposed to song or no-song treatments suggests that other kinds of social cues may be more potent than song recordings alone. Given the study design, it is difficult to determine what cues the live male provided that stimulated females. As only one of the five females in the chamber could see the male, it is unlikely that visual cues played a role. Instead, vocal interactions between the live male and females, or variation in how the male presented his song (time, duration, sequential presentation of vocalizations, degree of stereotypy) compared with the recording seem more likely explanations (Tramontin et al., 1999). Importantly, this includes the possibility that the female birds in this treatment group were responding behaviorally to the live male and this behavioral response (including posture, or the females' own vocalizations) led to the enhanced effect. Such female ‘self-stimulation’ has been demonstrated in other systems (Cheng, 1992).

Given the absence of an effect of recorded male song on gonadal development in either subspecies, the effect on peak molt in migrants is especially interesting. While some studies have reported the effects of non-photic cues on prebasic molt (Wingfield et al., 2003), very little is known about how they can affect pre-alternate molt (Wingfield and Silverin, 2009). One possibility is that song cues actually stimulate molt and reproductive preparation in migrants, but the experiment did not continue long enough to observe the effect on ovarian development (though see the discussion above on photoperiod and social cue interactions). It is also possible that it is easier to detect the effects of a song cue on molt than on ovarian development and this raises interesting questions as to how regulation differs between these related but distinct processes. There may be something unique about the cues regulating molt, distinct from other pre-migratory processes, as we did not detect a difference in response of pre-migratory fattening (measured as a mass index) to a song cue in migrants.

While our study does not support specialization in response to a recorded male song stimulus between migrants and residents, it does not preclude the possibility of specialization in the use of other social cues or in cues like temperature and photoperiod. Indeed, our findings that all aspects of ovarian development and pre-migratory fattening (mass index score) differed between migrants and residents is consistent with common garden studies showing that migrants and residents respond differently to photoperiodic cues (Fudickar et al., 2016; Helm, 2009; Ramenofsky et al., 2017). This finding is also consistent with the adaptive specialization hypothesis, as additional work in stonechats suggests that specialization in photoperiodic response may be adaptive, instead of merely indicating a phylogenetic constraint (Helm, 2009). Two interesting questions that arise from this work are: (1) what kinds of cues are associated with adaptive specialization in seasonal timing mechanisms between groups?; and (2) what kinds of life history differences do we expect to be associated with adaptive specialization in seasonal timing mechanisms? For migrants and residents, much work has speculated that the response to photoperiod and climatological factors (like weather) may exhibit specialization (see Hahn and MacDougall-Shackleton, 2008) and findings in altitudinal migrants are consistent with this idea (Perfito et al., 2005). However, the kinds of cue use differentiation between migrants and residents may depend upon whether they ever experience the same environmental conditions and, if so, when during the annual schedule they experience those shared conditions (i.e. migrants and residents that share wintering grounds may experience different patterns of adaptive specialization in cue use from those of birds that share breeding grounds, or two species that never experience the same environment). Additionally, it is important to consider that social cues might not be the kind of cue that is used differently between migrants and residents. The null results in our study leave open both the possibility that (1) migrants and residents respond similarly to social cues and our recorded song presentation was insufficient to stimulate a response in either subspecies (consistent with the conditional plasticity hypothesis) or (2) migrants and residents are equally unresponsive to social cues generally (although the results of our live male presentation and work by Morton et al., 1985, make this possibility seem unlikely). One productive area for future inquiry may be to investigate differences in social cue responsiveness in birds with different patterns of social interactions over the course of the breeding and non-breeding seasons. For example, if birds have limited contact with potential mates during the non-breeding season (e.g. golden eagles), they may be unlikely to use social cues in reproductive preparation, especially during initial growth phases. In contrast, birds that live in large mixed-sex flocks during the non-breeding season (e.g. many passerines) may be more likely to utilize social cues during reproductive preparation.

### Conclusions

Understanding the similarities in how migrants and residents use and respond to environmental cues is important, as frequently these differences are used to underpin predictions about how they will respond to climate change (McNamara et al., 2011). While a large body of theory exists suggesting that migrants and residents should have differences in how they respond to non-photic cues, to our knowledge this is the first explicit test of that hypothesis. Explicit comparisons of how migrants and residents respond to different kinds of cues is an area with great potential for future research.

One important consideration arising from this study is whether migrants and residents should be expected to differ in their use of all non-photic cues. Much of the theory about differences in cue use between migrants and residents is driven by the assumption that non-photic cues on the wintering grounds are not informative of conditions on the breeding grounds for migrants. While this may often prove true for non-photic cues like temperature, food availability and precipitation, social cues are a fundamentally different kind of information. A more robust understanding of how social interactions change over the wintering period may reveal that social cues can prove similarly informative for migrants and residents even during initial preparation for breeding and other vernal life history stages.

Beyond understanding how migrants and residents differentially respond to cues, it is important to determine the mechanistic level at which cue–response system differences translate into differences in physiology and behavior. Migrant female dark-eyed juncos respond less to GnRH injections than resident females (Greives et al., 2016) and work in house sparrows suggests that exposure to males of varying reproductive states can influence neural GnRH-I and GnRH-II detected by immunocytochemistry (Stevenson et al., 2008). Our study suggests that differences in response to photoperiod are sufficient to cause differences in LH and ovarian development in migrant and resident white-crowned sparrows and that additional differences in GnRH production and release may drive this pattern upstream.

In sum, future opportunities should refine our understanding of cue use differences in migrants and residents, particularly in female birds. Our research suggests that there is more to know about how different kinds of cues (photoperiodic, weather and social) are similar and different and how their use is specialized across species. Additionally, much more remains to be understood about how the kinds of cues regulating different seasonal processes such as pre-alternate molt, pre-migratory fattening and gonadal recrudescence may be similar or different.
